# Visualization of spatial dose distribution for effective radiation protection education in interventional radiology: obtaining high-accuracy spatial doses

**DOI:** 10.1007/s13246-024-01479-w

**Published:** 2024-09-09

**Authors:** Yutaro Mori, Tomonori Isobe, Yasuwo Ide, Shuto Uematsu, Tetsuya Tomita, Yoshiaki Nagai, Takashi Iizumi, Hideyuki Takei, Hideyuki Sakurai, Takeji Sakae

**Affiliations:** 1 Institute of Medicine, University of Tsukuba, 1-1-1 Tennodai, Tsukuba, Ibaraki 305-8575 Japan; 2 Graduate School of Comprehensive Human Sciences, Degree Programs in Comprehensive Human Sciences, Doctoral Program in Medical Sciences, University of Tsukuba, 1-1-1 Tennodai, Tsukuba, Ibaraki 305-8575 Japan; 3 Graduate School of Comprehensive Human Sciences, Degree Programs in Comprehensive Human Sciences, Master’s Program in Medical Sciences, University of Tsukuba, 1-1-1 Tennodai, Tsukuba, Ibaraki 305-8575 Japan; 4 Department of Radiation Oncology and Proton Medical Research Centre, University of Tsukuba Hospital, 2-1-1 Amakubo, Tsukuba, Ibaraki 305-8576 Japan; 5 Department of Radiological Technology, Tsukuba International University, 6-20-1 Manabe, Tsuchiura, Ibaraki 300-0051 Japan; 6 Department of Radiology, University of Tsukuba Hospital, 2-1-1 Amakubo, Tsukuba, Ibaraki 305-8576 Japan; 7 Quantum Life and Medical Science Directorate, National Institute for Quantum Science and Technology, 4-9-1 Anagawa, Inage-ku, Chiba 263-8555 Japan

**Keywords:** Education and training, Radiation visualization, Occupational exposure, Monte Carlo simulation, Three-dimensional dose distribution

## Abstract

In recent years, eye lens exposure among radiation workers has become a serious concern in medical X-ray fluoroscopy and interventional radiology (IVR), highlighting the need for radiation protection education and training. This study presents a method that can maintain high accuracy when calculating spatial dose distributions obtained via Monte Carlo simulation and establishes another method to three-dimensionally visualize radiation using the obtained calculation results for contributing to effective radiation-protection education in X-ray fluoroscopy and IVR. The Monte Carlo particle and heavy ion transport code system (PHITS, Ver. 3.24) was used for calculating the spatial dose distribution generated by an angiography device. We determined the peak X-ray tube voltage and half value layer using Raysafe X2 to define the X-ray spectrum from the source and calculated the X-ray spectrum from the measured results using an approximation formula developed by Tucker et al. Further, we performed measurements using the “jungle-gym” method under the same conditions as the Monte Carlo calculations for verifying the accuracy of the latter. An optically stimulated luminescence dosimeter (nanoDot dosimeter) was used as the measuring instrument. In addition, we attempted to visualize radiation using ParaView (version 5.12.0-RC2) using the spatial dose distribution confirmed by the above calculations. A comparison of the measured and Monte Carlo calculated spatial dose distributions revealed that some areas showed large errors (12.3 and 24.2%) between the two values. These errors could be attributed to the scattering and absorption of X-rays caused by the jungle gym method, which led to uncertain measurements, and (2) the angular and energy dependencies of the nanoDot dosimetry. These two causes explain the errors in the actual values, and thus, the Monte Carlo calculations proposed in this study can be considered to have high-quality X-ray spectra and high accuracy. We successfully visualized the three-dimensional spatial dose distribution for direct and scattered X-rays separately using the obtained spatial dose distribution. We established a method to verify the accuracy of Monte Carlo calculations performed through the procedures considered in this study. Various three-dimensional spatial dose distributions were obtained with assured accuracy by applying the Monte Carlo calculation (e.g., changing the irradiation angle and adding a protective plate). Effective radiation-protection education can be realized by combining the present method with highly reliable software to visualize dose distributions.

## Introduction

### Lowering the eye lens dose limit

Publication 118 (Pub. 118) [1], which was published by the International Commission on Radiological Protection (ICRP) in August 2012, significantly contributed to the changes made to the eye lens dose limit. The dose limit for cataract, lens opacity, and dose limits for acute, fractionated, and chronic exposures were lowered significantly to a uniform dose limit of 0.5 Gy based on latest scientific evidence.

The recommendations in Pub. 118 resulted in an international movement for incorporating the changes into radiological protection systems. Further, in 2013, the International Atomic Energy Agency (IAEA) TECDOC 1731 proposed the “implications of new lens dose limits for the protection of radiation workers” and introduced new dose limits [2] According to TECDOC 1731, healthcare workers engaged in interventional radiology (IVR) and nuclear medicine (radiation workers) specifically require eye lens protection. Many international organizations and national laws reviewed the eye lens dose limits, drawing significant social attention on these issues.

### Problem of eye lens exposure in the medical field

The revision of the eye lens dose limit led to several issues in the fields of X-ray fluoroscopy and IVR performed for radiological diagnosis and treatment. Physicians, nurses, and radiological technologists from the radiology department are typically involved in performing X-ray fluoroscopy and IVR. Besides these radiology workers, physicians, especially those who do not specialize in radiation (e.g., orthopedic surgeons, cardiologists, brain surgeons, and gastroenterology surgeons), often perform these procedures, exposing themselves to particularly high radiation doses. Given this context, several fact-finding surveys and studies on protective measures have been reported [3–5].

The principal guidelines for avoiding radiation injury in IVR are given in ICRP Pub. 85 [6]. This report has led to the publication of many research reports on occupational exposure in IVR. Chida has compiled a detailed survey of occupational exposure in IVR [7] and a report on dose reduction [8]. Vano et al. reported on optimization strategies in IVR [9]. Several studies focusing on the lens of the eye have precisely evaluated the exposure of the lens of the eye of medical staff in IVR [10, 11]. Matsubara et al. measured the exposure of the lens of the eye of physicians in IVR and analyzed its association with an increased risk of cataract development [12]. Radiation exposure associated with endoscopy and computed tomography (CT) examinations, when working indoors during irradiation, has also been reported [13–16]. Therefore, controlling and reducing radiation exposure to radiation workers in the medical field is an important issue. To address this issue, extensive research has been conducted on the development and use of new radiation protective equipment [17–20]. In particular, protective eyewear has been shown to be effective in reducing lens exposure doses [18, 20]. Despite these protective measures, the problem of occupational exposure among health care workers remains unresolved. One approach to resolving this problem is employing radiation-protection education and training. For example, Vano et al., discusses the importance of radiation-protection education and training in diagnostic radiology and IVR [6, 9, 11, 12, 21]. Further, Rehani et al. states that radiation protection in fluoroscopy-guided procedures performed outside the diagnostic imaging department requires an extensive understanding of protecting against X-ray scattering [22].

### Improvement of educational effectiveness through radiation visualization and current issues

Radiation-protection education can be provided in a limited amount of time through the knowledge-telling approach [23]. However, the learning retention rate is ~ 5% for this approach [24] which is significantly lower than that required for educational effectiveness. In contrast, the learning retention rate for the visual and hands-on experience approach is ~ 75%, [24] indicating a clear difference in the educational effectiveness between the two approaches. This result is consistent with the study reported by Kato, which indicated that people obtain 80% of their information visually [25]. Monte Carlo simulations can be used to effectively visualize radiation generated by X-ray generators in radiation-protection education to educate and alert healthcare workers [26, 27]. Such simulation-based educational learning approaches can effectively improve the competence of healthcare workers without radiation exposure to patients [28]. However, these reported studies do not focus on improving or validating the calculation accuracy of spatial dose distributions. The visualization and education conducted hypothetically using calculation results that deviate considerably from reality can pose a serious problem of fostering erroneous comprehension by learners.

This study presents a method to maintain a high level of calculation accuracy for the spatial dose distribution generated by X-ray fluoroscopy and IVR equipment using Monte Carlo simulation and establish a method for the three-dimensional visualization of radiation using the obtained calculation results.

## Materials and methods

### High-accuracy calculation of spatial dose distribution

#### Monte Carlo simulation

The Monte Carlo code particle and heavy ion transport code system (PHITS) Ver. 3.24 [29] was used to design the angiography room used for reproducing the spatial dose distribution generated by the angiography device (INFX-8000 V/J4, Canon Medical Systems Corp., Tochigi, Japan). The structure of the target (radiation generating unit) is the most important factor when reproducing a radiation generator using Monte Carlo simulation; however, the structure of the target is confidential manufacturer information and cannot be reproduced via Monte Carlo simulation. Therefore, we use the X-ray energy spectrum of the device obtained through a combination of actual measurements and calculations and set it as the radiation source for the Monte Carlo calculations.

**Fig. 1:**
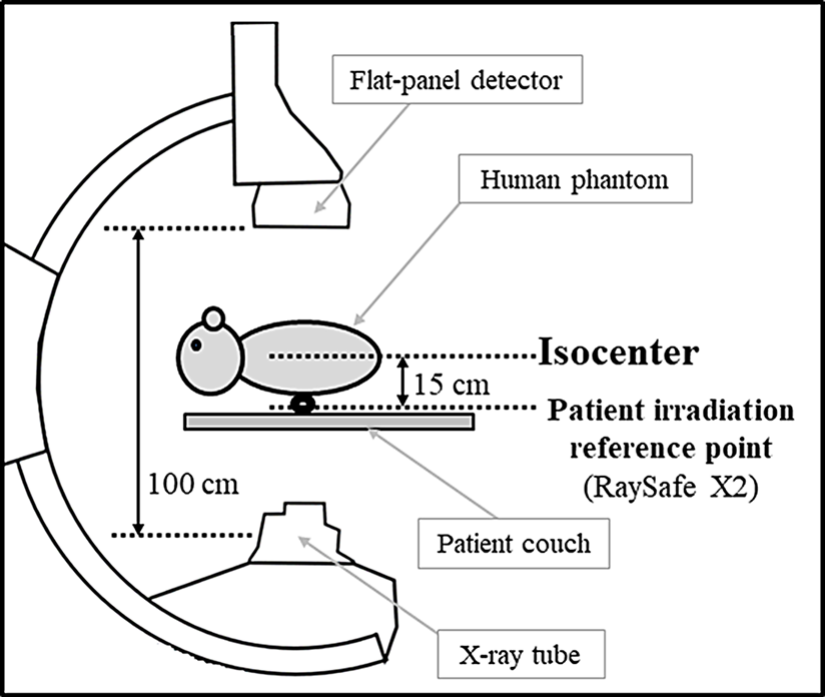
Geometric arrangement of the angiography device used for X-ray spectral evaluation. The RaySafeX2 was positioned 15 cm below the isocenter to acquire the peak tube voltage and half value layer output from the angiography device

The measurement geometry for calculating the energy spectrum is shown in Fig. 1. Irradiation was performed in the posteroanterior direction (assuming under-tube fluoroscopy), the distance between the X-ray tube focus and X-ray receiver was 100 cm, and the height of the patient’s bed was 100 cm from the floor. A human body-equivalent thoracoabdominal land phantom (The Phantom Laboratory, Inc., Salem, New York) was placed as a scatterer at the center of the irradiation field on the patient couch. The semiconductor X-ray detector (Raysafe X2, Unfors RaySafe AB, Hovås, Sweden) was placed at the patient irradiation reference point 15 cm below the isocenter. The irradiation field was set at 19.8 cm x 19.8 cm, and irradiation was performed with a tube voltage of 70 kV and tube current of 130 mA set by automatic exposure control. The peak tube voltage and half value layer output from the angiography device were determined using Raysafe X2 under these conditions. The angular dependence, energy dependence, stability, reproducibility, etc., of the Raysafe X2 are as shown in its specification sheet [30]. Additionally, the Raysafe X2 was calibrated using the substitution method according to JIS Z4511, with standards traceable to Japanese national standards.

Based on the obtained peak tube voltage and half value layer, the X-ray energy spectrum was calculated using the approximation formula of Tucker et al. [31]. Calculations were performed using the software X-Tucker-4 [32], which incorporates the algorithm developed by Tucker et al. Monte Carlo simulations were performed using the obtained energy spectrum data to calculate the spatial dose distribution in an angiography room (780 cm, long-axis direction; 560 cm, short-axis direction; and 300 cm, height). For the calculation, the photon cut-off energy was set at 5 keV, and the spatial dose was calculated by dividing the room space every 1 cm. The number of histories was set at 1 × 10^10^, and the calculations were performed until the statistical error of the Monte Carlo calculation results was less than 5%.

#### Verification of the Monte Carlo calculation accuracy based on actual measurements

**Fig. 2:**
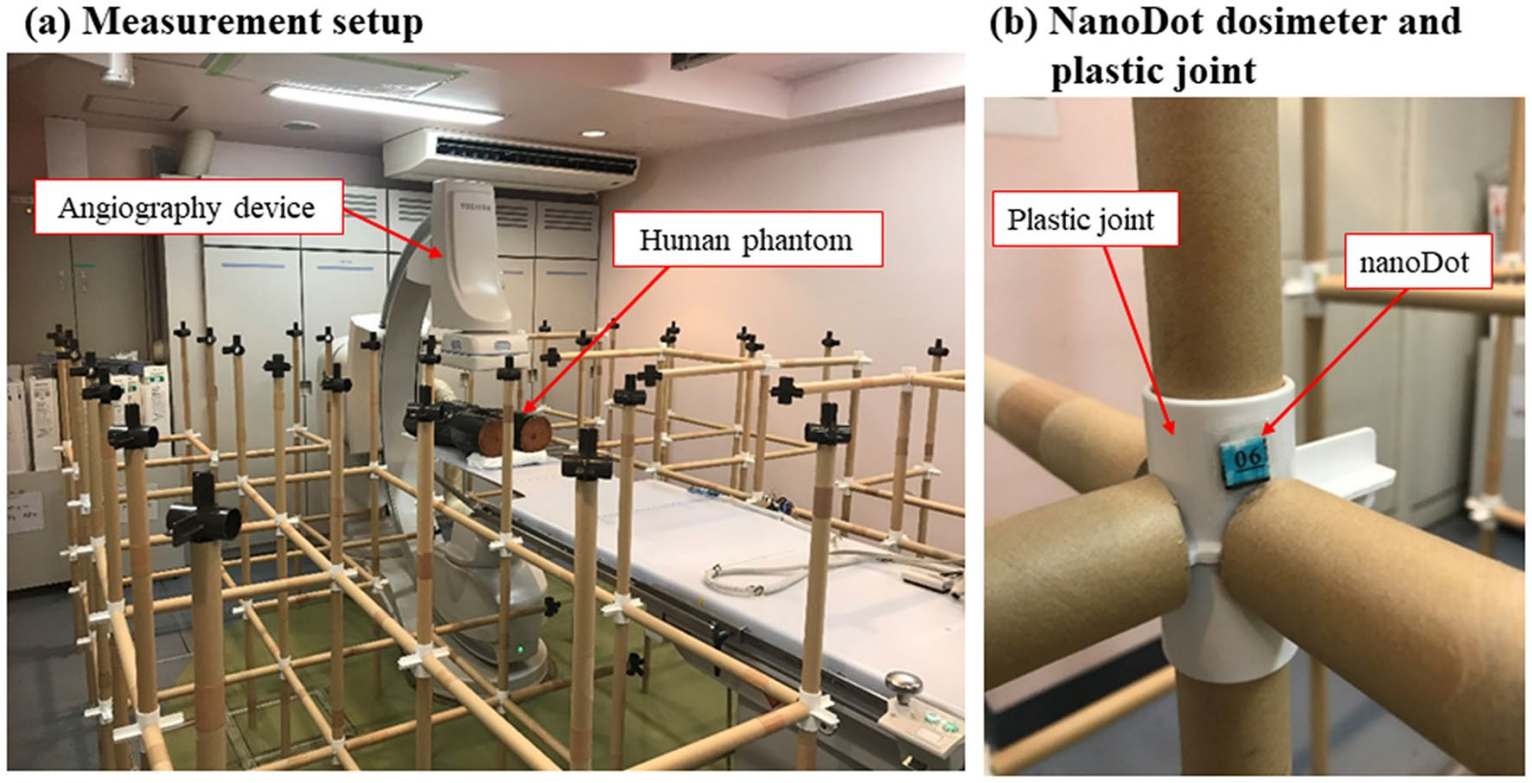
Spatial dose measurement. The spatial dose measurement setup in the angiography room is shown in Fig. 2. The spatial dose was measured at 49 measurement points set up using the jungle-gym method

**Fig. 3:**
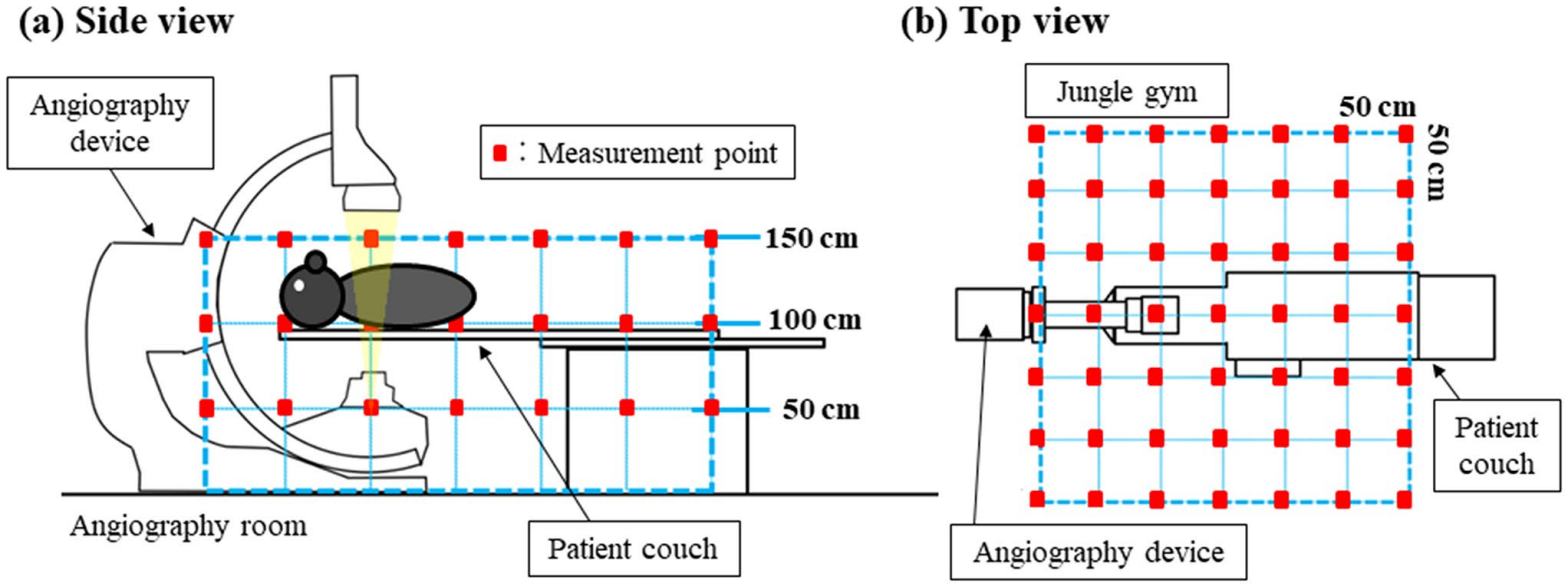
Measurement points set using the jungle-gym method. The measurement points set using the jungle-gym method are shown in Fig. 3. The measurement points were set at 50, 100, and 150 cm above the floor. A nanoDot OSL dosimeter was attached to the measurement points

The accuracy of the Monte Carlo calculation was verified by performing actual measurements using a measurement system (jungle-gym method) [33] that consists of paper pipes and plastic joints placed in a grid under the same irradiation conditions as in i). Figure 2 (a) illustrates the measurement setup when using the jungle-gym method. Paper pipes (length = 50 cm) and plastic joints (Fig. 2 (b)) were combined and assembled in a three-dimensional grid around the angiography device. The measurements were performed at heights (z-axis) of 50, 100, and 150 cm from the floor (Fig. 3 (a)). The total number of measurement points on the plane was 49: 7 points on the x-axis and 7 points on the y-axis at 50 cm intervals (Fig. 3 (b)). The long- and short-axes of the bed at the isocenter were defined as the x- and y-axes, respectively, and the jungle gym was assembled in a grid manner. The points where jungle-gym joints could not be assembled because of the structure of the examination room or equipment, such as the legs of the patient couch or the C-arm of the angiography device, were excluded from the measurement points as they were deemed unmeasurable. An optically stimulated luminescence (OSL) dosimeter, nanoDot (Landauer, Inc., Glenwood, IL), is used as the compact dosimeter in the study (Fig. 2 (b)). The nanoDot dosimeters were used in conjunction with the microStar reader (Landauer, Inc., Glenwood, IL) as supplied by Landauer. Moreover, an optical filter was added to microStar to enable measurements as low as 0.001 mGy and improve measurement accuracy by recording three readings for calculating the average value [34]. The evaluation of air kerma *D* (mGy) by the OSL dosimeter was calculated using the following equation. In this equation, the post-irradiation reading (Count) is *C*_post_, the pre-irradiation reading (Count) is *C*_pre_, the calibration constant of the reading device (Count/mGy) is *CF*, and the sensitivity of each element is *k*_s_.

$$\:D\left(mGy\right)=\frac{{C}_{post}-{C}_{pre}}{CF\times\:{k}_{s}}$$

The OSL dosimeter used in this study has a wide range of dose linearity in dosimetry, with ± 10% at 10 µGy for both X-rays and γ-rays, and within ± 5% at higher doses [35]. Furthermore, the energy response is sufficient for dosimetry, with ± 10% from 20 keV to 1 MeV [35]. The nanoDot used to measure the air dose was calibrated by Nagase-Landauer, Ltd. (Osaka, Japan) on a phantom with 80 kVp X-rays.

The attachment of the nanoDot dosimeter was adjusted towards the X-ray tube on the plastic joint (i.e., the measurement point). The experimental geometry and irradiation conditions were the same as the Monte Carlo simulation previously described, and the fluoroscopy pulse rate was set at 15 pulses/s for 10 min and converted from *D* to dose rate (mGy/h) at each measurement point.

### Three-dimensional visualization of radiation

Three-dimensional spatial dose distributions based on the created data were displayed using ParaView (version 5.12.0-RC2) [36]. The spatial dose distributions of the direct X-rays and the spread of the scattered X-rays were displayed separately to improve the effectiveness of the radiation-protection education.

## Results

### High-accuracy calculation of spatial dose distribution

**Fig. 4:**
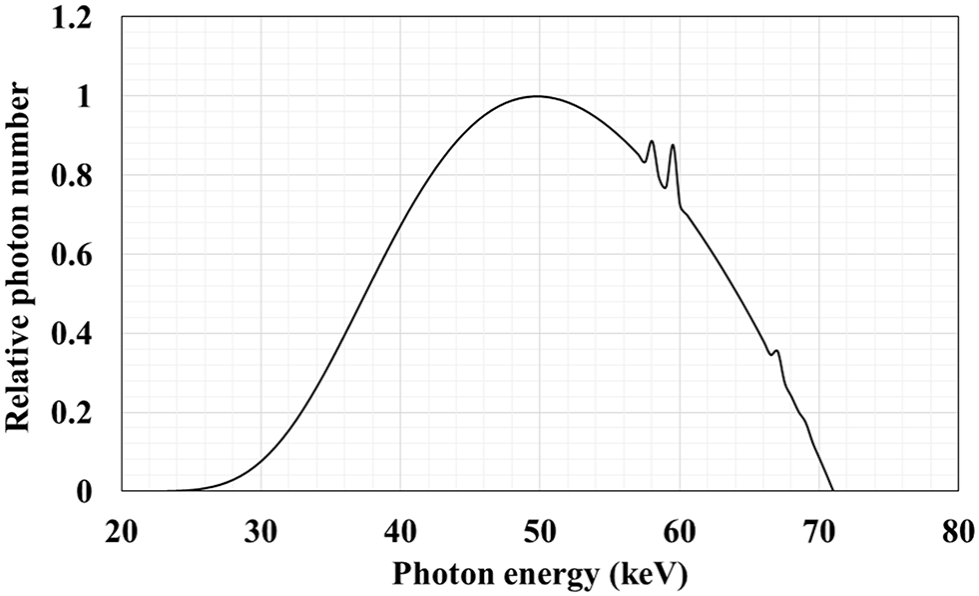
X-ray energy spectrum from the angiography device obtained by the approximation formula (Tucker et al.) for the calculation of the X-ray spectrum. The X-ray energy spectrum calculated under the same conditions as the actual measurement is shown in the figure. A continuous spectrum with a maximum energy of 70.8 keV was obtained

The peak tube voltage of the primary X-rays determined by Raysafe X2 under actual fluoroscopic conditions was 70.8 kVp and that of the half value layer was 6.06 mmAl. Further, the target angle of the X-ray tube focus in the angiography device and copper-added filter thickness (0.2 mm) used selectively during X-ray fluoroscopy were input to X-Tucker-4 for calculating the X-ray spectrum. The X-ray energy spectrum calculated from the approximation formula of Tucker et al. is shown in Fig. 4. We obtained a continuous energy spectrum with a peak voltage of 70.8 keV. Based on the spectral data, the radiation source was defined in PHITS to perform the Monte Carlo calculation.

**Fig. 5:**
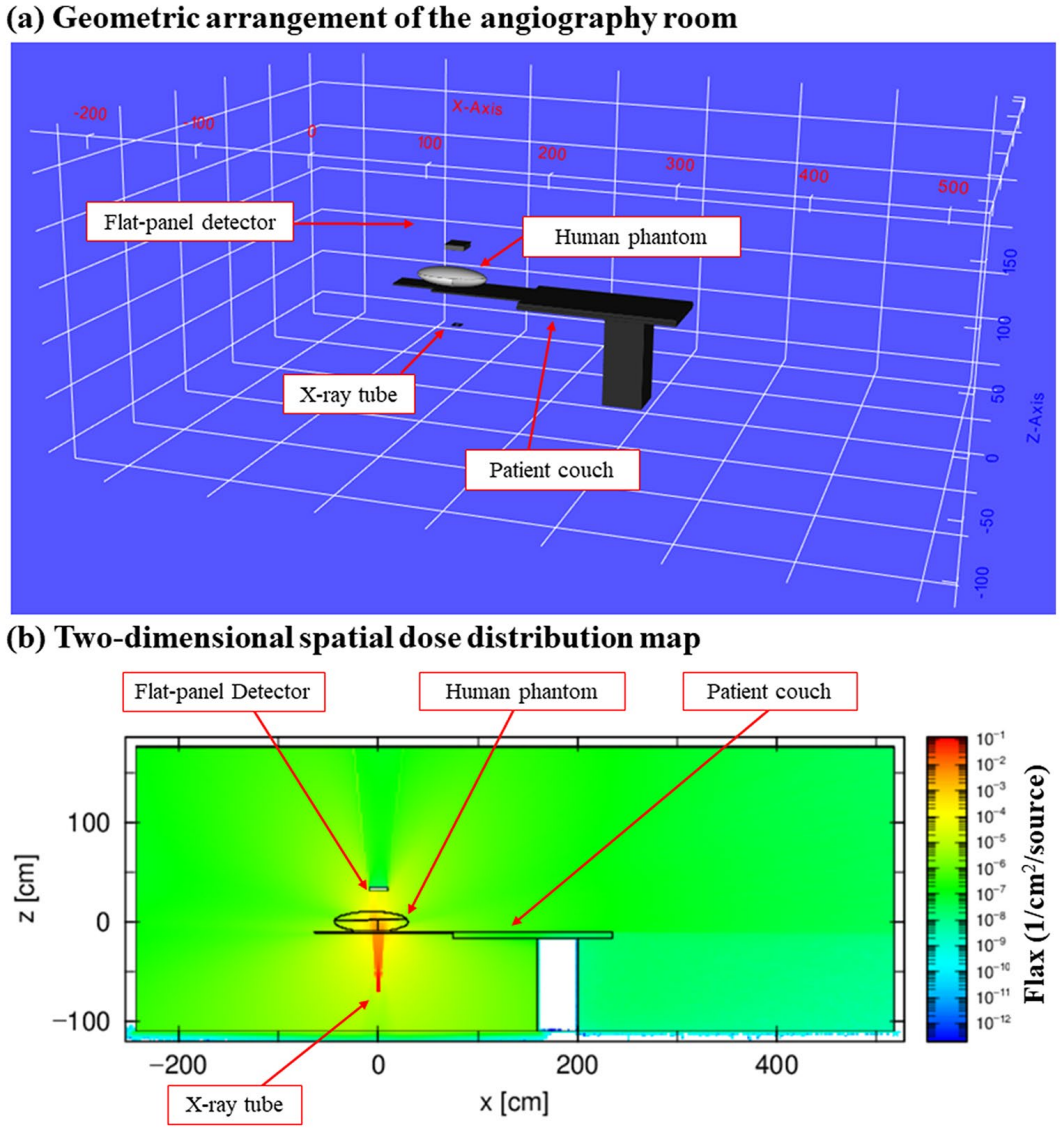
Geometry of the angiography room reproduced by (**a**) PHITS and (**b**) the two-dimensional spatial dose distribution. The reference (0) of the coordinate system is the long-axis direction of the patient couch, the X-ray tube source position is the short-axis direction, and the height direction is the isocenter

Figure 5 (a) shows the geometric arrangement of the angiography room created by PHITS. Figure 5 (b) shows the two-dimensional spatial dose distribution map in the cross-section parallel to the axis of the imaging room and passing through the X-ray tube (Y-axis: 0) among the three-dimensional spatial dose distributions of the angiography room obtained by the Monte Carlo calculation. The primary X-ray beam was emitted from the source in the vertical upward direction and entered the X-ray receiver in the form of a fan beam.

**Fig. 6:**
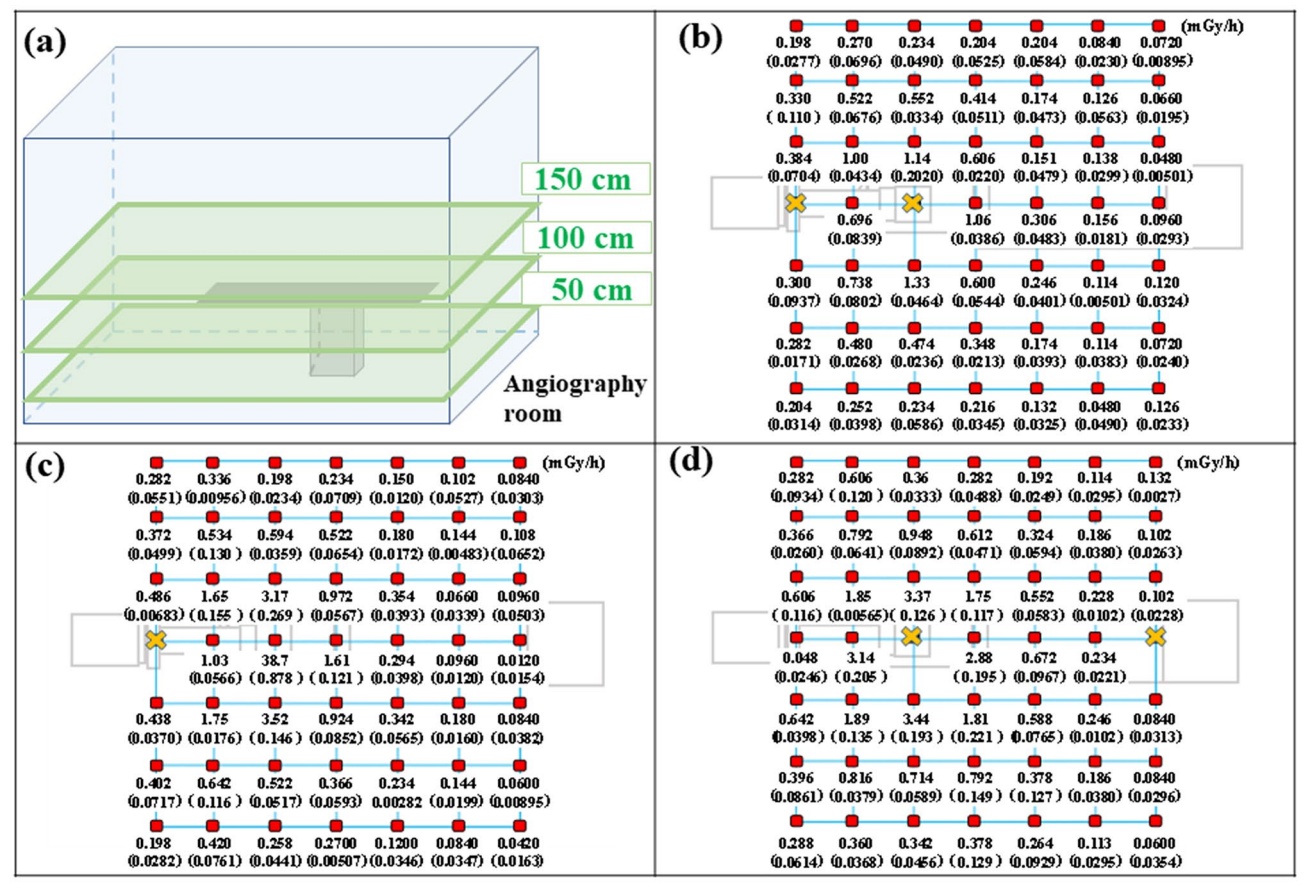
Measurement results of the spatial dose rates in the angiography room. (**a**) Cross-section of the measurement. (**b**)–(**d**) Measurement results at heights of 150, 100, and 50 cm from the floor, respectively. The unit of measurement is mGy/h. The values in parentheses indicate the standard deviation

Spatial doses measured at heights of 50, 100, and 150 cm from the floor obtained by actual measurements are shown in Fig. 6. The vertical axis represents the spatial dose rate (mGy/h) at each measurement point, and the horizontal axes represent the position of the nanoDot dosimeter (x-axis, − 100–200 cm; y-axis, − 150–150 cm) with the position of the X-ray tube as 0. The spatial dose rate at each measurement height was high near the X-ray tube (x-axis, 0 cm; y-axis, 0 cm) and tended to decrease with an increase in the distance from the X-ray tube. Further, compared to heights of 50 (Fig. 6 (d)) and 100 cm (Fig. 6 (c)) from the floor, the spatial dose rate at a height of 150 cm (Fig. 6 (b)) decreased markedly.

**Fig. 7:**
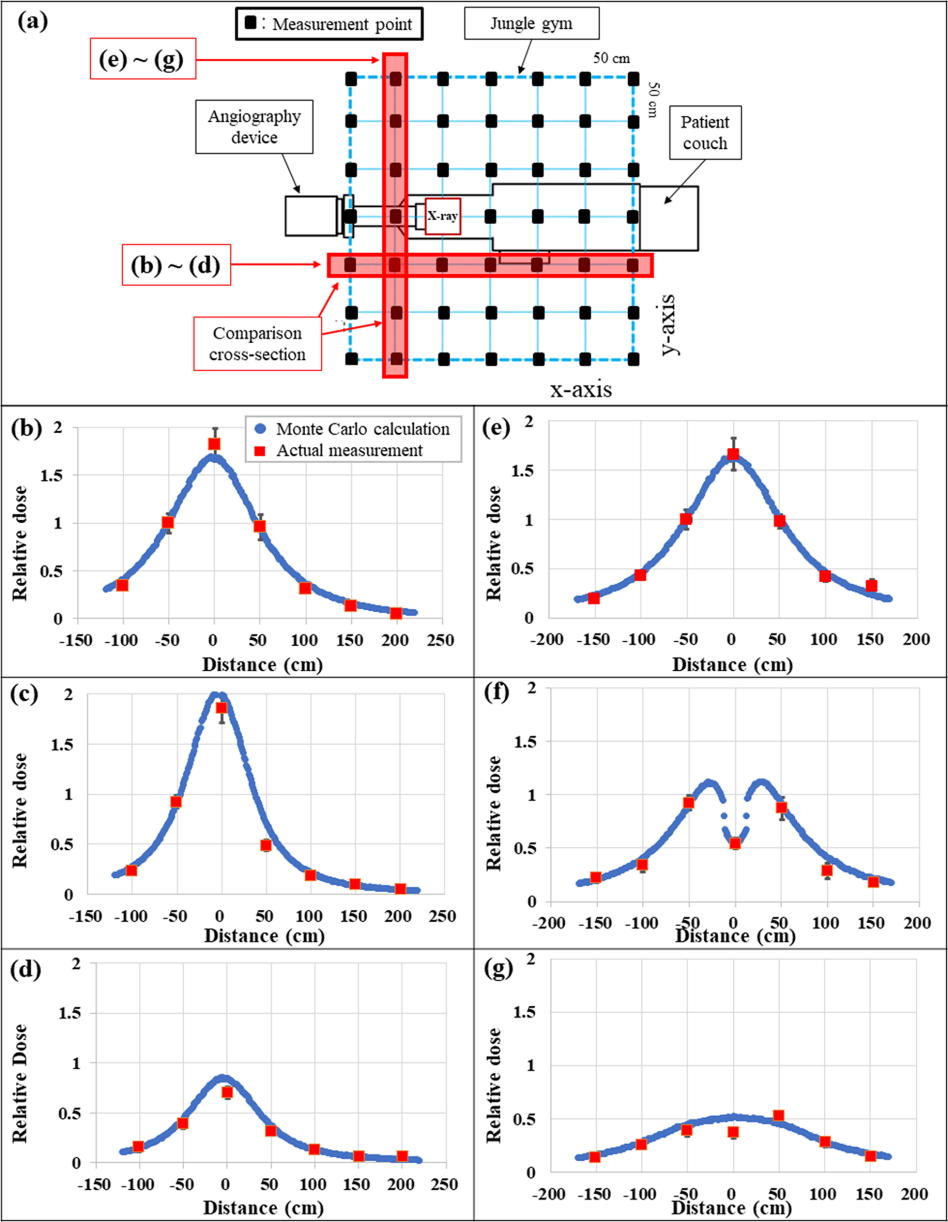
Relative spatial dose at each measurement height. (**a**) Cross-section comparison between the actual measurement and MC calculation, (**b**) Angiography room x-axis, height from floor: 50 cm, (**c**) Angiography room x-axis, height from floor: 100 cm, (**d**) Angiography room x-axis, height from floor: 150 cm, (**e**) Angiography room y-axis, height from floor: 50 cm, (**f**) Angiography room y-axis, height from floor: 100 cm, (**g**) Angiography room y-axis, height from floor” 150 cm

Figure 7 shows compares the results of the spatial dose distributions obtained by PHITS and through actual measurements. Cross sections close to the radiation source (X-ray tube) and where a large number of measurement points can be secured continuously (excluding cross sections where measurement points cannot be secured due to interference with the device or phantom) were used for comparison. Figure 7 (a) shows the row and column for the measurement points. Figure 7 (b)–(g) show the comparison between the measured and Monte Carlo calculated values for the cross section parallel to the long- and short-axes of angiography room, respectively, and 50 cm away from the X-ray tube.

Both calculated and measured values were normalized at the intersection of the two cross-sections (x, y, z) = (− 50, − 50, 50). The comparison results indicated that the two cross-sections were consistent within a 10% error overall, although deviations exceeded 10% for (x, y, z) = (0, − 50, 150) (the 0 cm distance point in Fig. 7 (d)) and (x, y, z) = (− 50, 0, 150) (the 0 cm distance point in Fig. 7 (g)), whose errors were 12.3 and 24.2%, respectively.

### Three-dimensional visualization of radiation

**Fig. 8:**
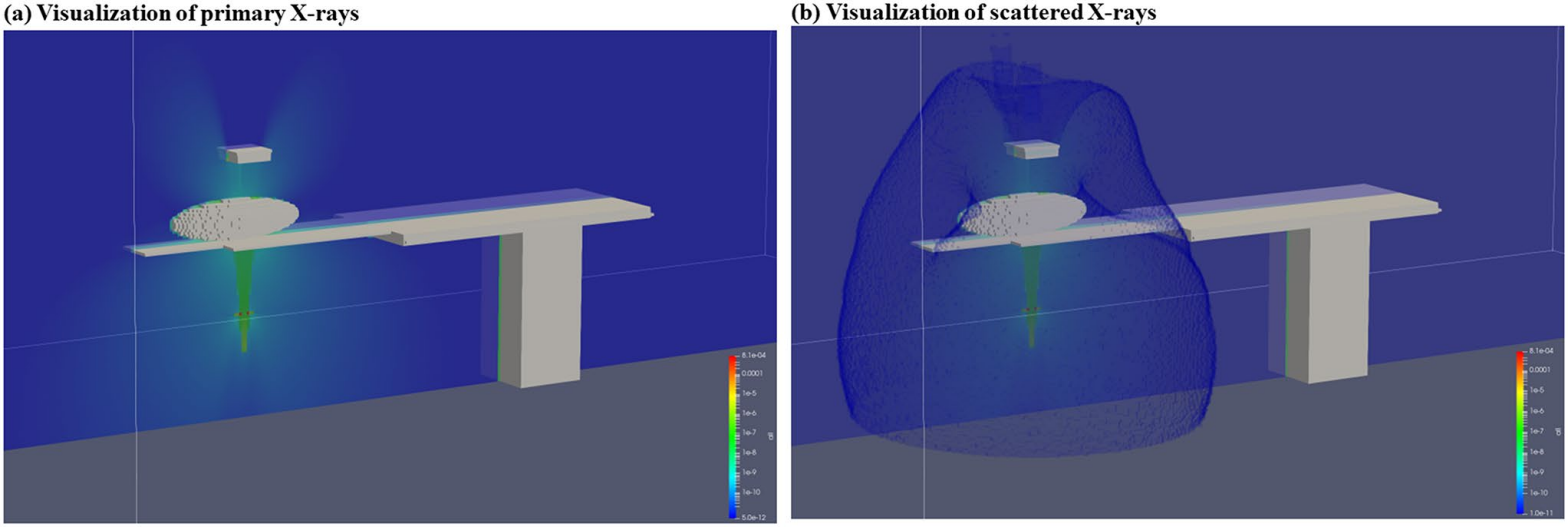
Radiation visualization using the ParaView. (**a**) Spread of primary X-rays and (**b**) spread including scattered X-rays

The Monte Carlo calculation results obtained in this study enable us to three-dimensionally visualize radiation using ParaView (Fig. 8). Figure 8 (a) and (b) show the direct and scattered X-rays, respectively, from the X-ray tube.

## Discussion

### Validity of spatial dose distribution by Monte Carlo calculation

**Fig. 9:**
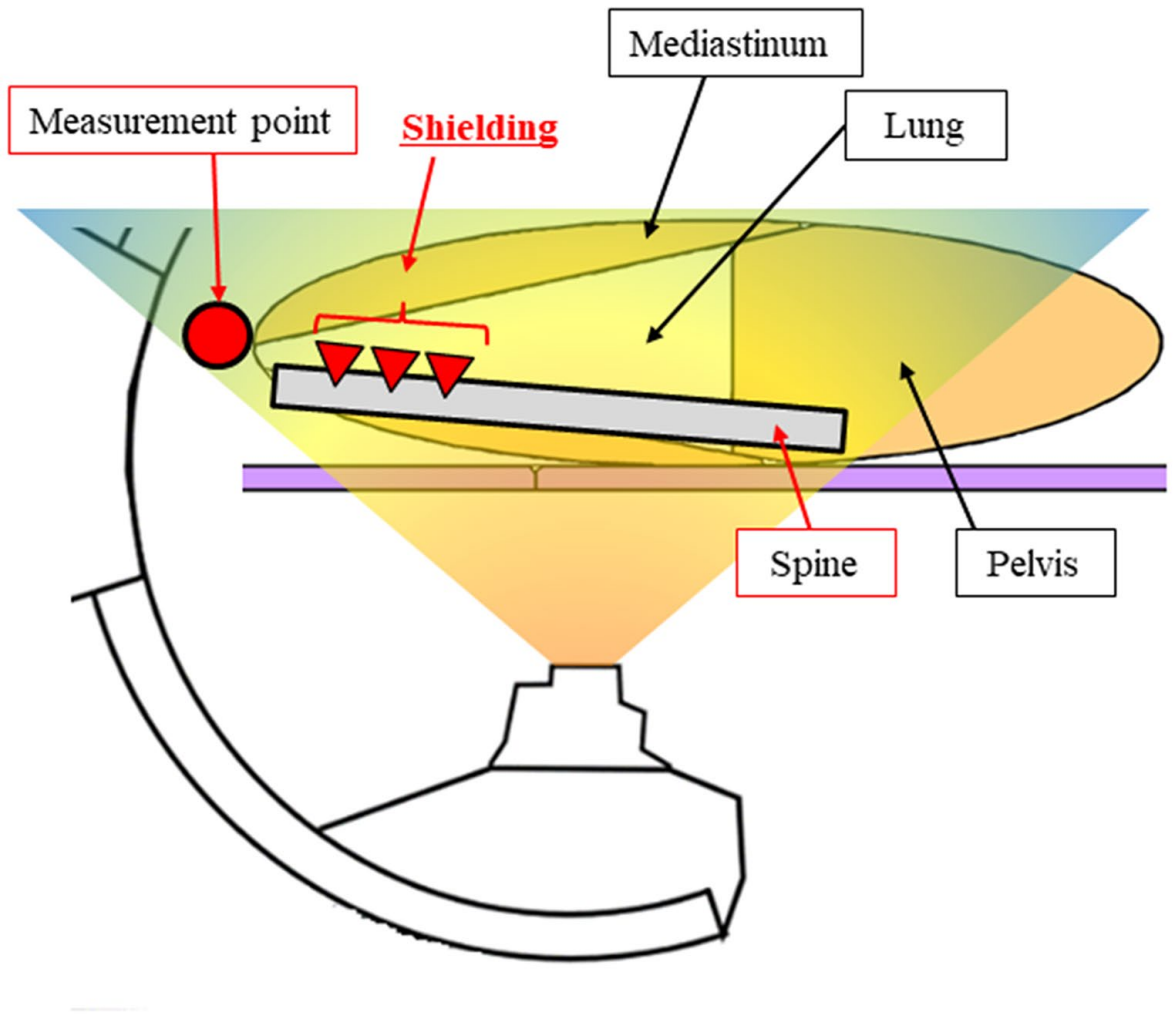
X-ray shielding by the spine

Comparing the measured and Monte Carlo calculated spatial dose distributions revealed that dose distributions at measurement points (Fig. 7 (b), (e)) at a height of 50 cm from the floor (closest to the height of the under-tube-type X-ray tubing) were consistent within 10%. The height of 50 cm from the floor had no structure directly shielded from the X-rays emitted from the X-ray tube, and therefore, the comparison results were based on the attenuation caused by the distance from the X-ray tube, which supported the high accuracy of the X-ray energy spectrum defined in this study. Further, the characteristic concavity near the horizontal axis 0, as shown in Fig. 7 (f), may have been caused by attenuation because of the spine structure of the phantom (Fig. 9). A similar trend was confirmed for both the actual measurement and the Monte Carlo calculation, thereby providing a factor to prove the high-accuracy calculation. In addition, as shown in Fig. 7 (g), the scattered X-rays were mixed sufficiently on the downward side, confirming that the effect on the spine was mitigated.

The spine of the phantom was located on the beam axis of the measurement point, and the attenuation caused by the high-density spine was confirmed for both the measurement and the calculation.

For the two points, (x, y, z) = (0, − 50, 150) and (x, y, z) = (− 50, 0, 150), the case where the errors between the actual measurement and Monte Carlo calculation exceeded 10% can be attributed to the two causes in the actual measurement: (1) “Measurement uncertainty attributable to the jungle-gym method” and (2) “Measurement uncertainty using the nanoDot dosimeter.” These two causes are discussed below.

#### Jungle-gym method

The jungle-gym method uses paper pipes and plastic joints to create the grid. Nakamura et al. reported that direct X-ray irradiation to these components can affect measurements by a maximum of 20% [37]. Further, the farther away the measurement point is from the X-ray tube, the more structure components of the jungle gym are present in between, which can cause these effects to become large. The two points where the errors exceeded 10% were highly likely to be influenced by the structural components because the height from the floor was 150 cm and the distance from the X-ray tube was long. The measurement uncertainty caused by the jungle-gym components at each measurement point is calculated. This is a limitation when comparing the Monte Carlo calculation with the actual measurement.

#### nanoDot

The nanoDot has a very thin detector element of 0.1 mm and small directional characteristics [33]. However, an angular dependence was observed, and the magnitude of the angular dependence varies with energy. Previous studies that use the nanoDot dosimeter reported that the maximum angular dependence was 70, 40, 11, and 5% for the energy ranges of mammography [38], general radiography [38], CT [39], and radiation therapy [40], respectively. The tube voltage used in this study was 70 kVp, which is the condition closest to the 40% reported for general radiography. In this study, the relationship between the angular dependence of the measurements and the direction of each element of the nanoDot for the source center varies greatly based on the measurement point considered in the jungle gym method. If the distance from the X-ray tube is sufficiently long, the angle with the X-ray tube does not change significantly with changes in the measurement point with a 50 cm grid spacing. However, when the distance between the X-ray tube and a measurement point is close, the angle with the X-ray tube is noticeably different even with the same 50 cm movement of the measurement point.

Another error is caused by nanoDot’s calibration method. The nanoDot used in this study was calibrated on a phantom using 80 kVp X-rays and converted to a dose. However, when the nanoDot is affixed to the jungle gym as in this study, the effect of backscattering is different, and this can cause uncertainty in the measured values because of the calibration method. Further, there could be uncertainty in the reading of microStar. For nanoDot measurements in this study, the average of three measurements was used as the actual measured value. The variation counts of the measured values were larger when the scattered doses were smaller, indicating an ~ 10% variation at the measurement points far away from the X-ray tube. The measurement error caused by nanoDot was consistent with the manufacturer’s nominal value. Thus, we can say that the measurement accuracy in this study was reasonable [41].

Based on the abovementioned points, we discuss two points, (x, y, z) = (0, − 50, 150) and (x, y, z) = (− 50, 0, 150), where the error was large. At both measurement points, the angle between the nanoDot and X-ray tube focus was ~ 66°, which is considered a large measurement error because of the angle dependence. In this study, at this measurement point, the nanoDot was attached at a location where a plastic joint between the X-ray tube focus and nanoDot is unavoidable; therefore, the absorption of X-rays caused by the jungle-gym structure may have affected the measurement. Another possible error factor was that the shape of the human body phantom, as shown in Fig. 9, was not exactly reproduced. The human phantom reproduced by the Monte Carlo calculation in this study was a triaxial unequal ellipsoid, and the concave part on the back from the chest to the neck was reproduced; however, the convex part on the neck was not. The scattering and absorption of primary X-rays affected the measured values, and the values may have been smaller than the Monte Carlo calculation values. However, the Monte Carlo calculated spatial dose distribution was reproduced within the uncertainty of the measured values, and therefore, it can be considered as a dose distribution whose accuracy is guaranteed by the actual measurements.

### Three-dimensional visualization of radiation

We successfully visualized the spatial dose distribution with guaranteed calculation accuracy through the present procedures. Further, the direct and scattered X-rays can be displayed separately, which is significant because it enables protective education depending on either direct or scattered X-rays. Mixed reality technology has witnessed remarkable technological developments in recent years, including airborne haptics technology. Chida et al. used a pinhole camera to visualize radioactive sources for the radiation protection of physicians and staff in IVR [42]. Matsusaki et al. developed and evaluated an educational application to promote the proper use of ceiling-suspended radiation shielding screens in angiography rooms, reporting that the use of augmented reality (AR) technology improved educational effectiveness [43]. Combined with these technologies, experiential radiation protection education is possible, thereby leaving room for further development.

As indicated in the introduction, radiation protection is an important issue, especially for physicians and staff in IVR. Additionally, there are reports of inadequate radiation protection training for radiological technologists, who are specialists in handling medical radiation-generating devices [44]. To address this problem, ICRP113 states that radiation protection education and training are of utmost importance [21], and the development of education and training incorporating new technologies is still desired. This study will contribute to this effort.

## Conclusion

We established a method to obtain three-dimensional spatial dose distributions in an angiography room using Monte Carlo calculations and another method to verify the accuracy of these distributions using actual measurements. Three-dimensional spatial dose distributions cannot be easily obtained based on actual measurements because the measurement process is time consuming under all conditions. Although Monte Carlo calculations enable us to evaluate three-dimensional spatial dose distributions under all conditions, it is unclear if the calculation results are correct. The accuracy of the geometry of the Monte Carlo calculation can be guaranteed by performing this verification, and therefore, highly accurate three-dimensional spatial dose distributions can be obtained by applying the created files (changing the irradiation angle, adding protective plates, etc.). Combined with software that visualizes the obtained calculation results can ensure effective radiation protection education.

The datasets used and/or analyzed during the current study available from the corresponding author on reasonable request. 
